# An EANM position paper on the application of artificial intelligence in nuclear medicine

**DOI:** 10.1007/s00259-022-05947-x

**Published:** 2022-08-25

**Authors:** Roland Hustinx, Jan Pruim, Michael Lassmann, Dimitris Visvikis

**Affiliations:** 1 Division of Nuclear Medicine and Oncological Imaging, University Hospital of Liège & GIGA-CRC in vivo Imaging, University of Liège, Liège, Belgium; 2 Medical Imaging Center, Dept. of Nuclear Medicine and Molecular Imaging, University Medical Center Groningen, University of Groningen, Groningen, The Netherlands; 3 Department of Nuclear Medicine, University Hospital Würzburg, Würzburg, Germany; 4 LaTIM, INSERM, UMR 1101, University of Brest, Brest, France

**Keywords:** Artificial intelligence, Nuclear medicine, EANM

## Abstract

Artificial intelligence (AI) is coming into the field of nuclear medicine, and it is likely here to stay. As a society, EANM can and must play a central role in the use of AI in nuclear medicine. In this position paper, the EANM explains the preconditions for the implementation of AI in NM and takes position.

This paper describes the EANM position regarding the introduction of AI in the clinical field of nuclear medicine. This document complements the review article published by the EANM AI workgroup, which defines AI and describes the many applications of AI in image reconstruction, data correction, processing, and analysis as well as the potential contributions in all clinical aspects of nuclear medicine by Visvikis et al. [1]. Whereas AI is coming into the field of nuclear medicine just now, it has already become a full reality in our neighbouring speciality radiology. The US Centres for Medicare and Medicaid Services (CMS) officially granted their first ever reimbursement of a radiology AI algorithm in September 2020, which opened the door for broader coverage of AI imaging software in the clinic. Generally speaking, there used to be two views in the radiology world with regard to the impact of AI. The first one is rather optimistic and considers that AI will help strengthen the role of radiologists in the grand scheme of medical care [2]. In the other view, AI will take over tasks from radiologists, making it a foe rather than a complementary tool [3]. A recent survey indicates that there is a mild level of optimism among the radiological medical community as 62% do not believe the diagnostic radiologist’s job to be in danger due to AI [4], and greater acceptance is gaining ground in the community as it is increasingly viewed as a potential solution to the current shortage of radiologists, to improve the quality of the medical practice, as well as a means to reducing overall healthcare costs [5].

In nuclear medicine, we have barely started scratching the surface of these questions [6], perhaps considering that they will be resolved in due course without our direct intervention. AI applications in NM and radiology (but also other disciplines) share similarities, in particular the cross-sectional techniques used in hybrid imaging. Even though the introduction of AI in NM has been lagging behind, there is no reason to assume that the advantages, progress, solutions, and challenges encountered in other disciplines will not apply in NM. Furthermore, these developments are not limited to NM physicians alone. They will also extend to physicists, radiochemists, and radiopharmacists alike. Some aspects specific to nuclear medicine, i.e. the impact of short-lived isotopes on radiopharmaceutical preparation and patient scheduling, or the increased application of individual dosimetry in treatments, are likely to further enhance the potential impact of AI on our everyday practice.

For AI in NM to deliver on its promise, we need to identify those applications that we believe will benefit the most from the development of AI techniques. At the same time, we need to define how we want these developments to be evaluated and subsequently validated. Both these aspects need to be addressed before a technology can be adopted in routine clinical care. In addition, we jointly need to decide on the respective roles of humans and machines for each of the individual applications considered. Responsibilities in the face of rules and regulations, data privacy and security, and more generally, the ethics of AI in nuclear medicine, are also key issues that need to be defined and addressed. As a medical and scientific community, we need to ready ourselves for the arrival of AI, so that we remain agents of the changes rather than mere spectators on the sideline.

## Physics, clinic, and the need for thorough evaluation and extensive validation

It appears likely that the first routine applications of AI will be found in image reconstruction and processing (e.g. denoising, segmentation). While at first sight, such applications may seem fully under the control of physicists, they are not totally free of risk for patients and thus should also be watched with scrutiny by physicians. Image reconstruction with deep learning (DL) can generate errors and lead to artefacts and alterations that potentially have a clinical impact. True, the current state-of-the-art iterative reconstruction and associated data correction algorithms are also prone to certain artefacts. However, these are well known and easily recognisable. In contrast, machine learning algorithms—even the smartest—can be fooled by small alterations to the input data and completely mishandle the data leading to unpredictable artefacts [7, 8]. There is a continuum between the physics and the clinical applications of AI. Because of this interlinkage and the potential for error propagation, the need for strict validation at every stage should be emphasised. The general principle has been recognised by the EU with the risk-based approach of the European Artificial Intelligence Regulation, which aims at making healthcare solutions that present higher risks more safe, reliable, and trustworthy for healthcare professionals and patients [9].

Let us give an example: In [^18^F]FDG PET/CT imaging, the processes of identification, location, and segmentation of the lesions are often lumped into one, meaning that starting with the full volume of distribution of the tracer, i.e. the 3D PET image, the algorithm ends up with volumes of interest that each is recognised and qualified as a disease. For instance, Sibille et al. developed a DL algorithm using both the PET and the CT data that showed an 87.1% sensitivity and 99% specificity in classifying lung cancer patients and 88.6% localisation accuracy in the same population [10]. In patients with diffuse large B-cell lymphoma, the methodology proved to be predictive for both overall survival (OS) and progression-free survival (PFS) [11]. However, in this series and considering the segmentation task, the Sørensen–Dice coefficient, which is a measure of the similarity between the volumes determined by the physicians and by the DL algorithm, was only 0.65 in a research cohort and a mere 0.48 in a routine cohort. These results indicate that in its current form, in this population and contrary to the first impression, the DL solution is not very good at reproducing the physician’s performance in identifying and delineating disease, although it remains predictive of OS and PFS. This is most certainly related to the fact that the DL algorithm was not specifically trained for the purpose of accurately segmenting the 3D functional volumes but for characterising the volumes of interest. In addition, there are multiple DL algorithms using different architectures. Therefore, it should be borne in mind that it is highly unlikely that the same algorithm will be capable of performing multiple tasks with high precision. In the case of image segmentation, for example, U-NET has been shown to provide high accuracy results with different image scales, types, and resolutions [12]. This also holds true for functional PET images [13], where it shows similar or better performance than the best available semiautomatic segmentation algorithms developed over the past decade [14]. It has to be remembered that for a segmentation task, the ground truth is not known, and the results of any DL algorithm, therefore, are highly dependent on the annotations of human experts. The ‘gold standard’ is represented by the manually determined metabolic tumour volume, without any measure of interobserver or intra-observer reproducibility, or any further validation by other imaging methods or pathological confirmation. Hence, we do not know who is right or wrong in the discrepant cases. Is it the human “gold standard”, or is it the DL algorithm? Ultimately, the physician decides what to keep, what to correct, and what to discard, which means that we continue to accept inherent human flaws. On the other hand, if the product is designed to define the target volumes in radiation therapy planning, the question of who is right and who is wrong becomes more acutely relevant, as it will directly alter the planning target volume. And if the aim is to prognosticate or to evaluate the response to treatment, then clinical endpoints such as objective response or survival will take preeminence. It must therefore be emphasised that any single solution based on a given DL algorithm architecture should be considered within the context of the initial objective and the associated training that was performed. Results cannot be generalised to other situations without thorough validation.

## Explainability, causability, and the need for robustness

The concepts of explainability and causability have been developed by Visvikis et al., and it is clear that they are the foundations for trusting any AI application in clinical practice [1]. However, we are also convinced that more is needed. Current AI models are mostly static, in that they have been trained at a certain point in time using samples corresponding to a particular population and in a setup that was appropriately validated when the model was built. This validation should have involved the use of multiple datasets originating from centres external to the one in which the model was developed, in order to test its robustness on datasets that are as heterogeneous as possible (for example in terms of acquisition and reconstruction protocols). Furthermore, image standardisation and harmonisation techniques should be considered before widely applying such validated AI models. These static algorithms may also be subject to concept drifts. This means that even though a task is performed efficiently and reliably at first, it may no longer be the case when the patient population and associated treatment evolve or the imaging technique changes. Therefore, the algorithms should not stop learning, i.e. they should adapt in line with any modifications introduced in the data to be analysed. This is known as the continuous learning or continual AI concept [15]. The algorithm learns to learn and incrementally adapts to new characteristics found in the input data, constantly updating its feature selection to better fit its changing environment. Intuitively, we may realise the advantages of such a process. However, we should also realise that it should be associated with a constant “revalidation process”, as described above. Indeed, a catastrophic inference or forgetting may occur when extreme outliers wreak havoc in an autonomously relearning algorithm. To put it simply, even AI algorithms that are fully validated and trustworthy at the time of marketing and clinical implementation need to continuously undergo extremely stringent quality controls, comparable to phase IV post-marketing surveillance in the development of drugs. In this respect, the European Artificial Intelligence Act includes a solid post-marketing compliance and enforcement system, including market surveillance, post-marketing monitoring by the providers, a reporting system and reassessment in case of substantial changes to the AI system [16]. This further consolidates the premarket conformity assessment. The proposed European system will be designed to ensure the trustworthiness of AI systems over time.

## Role of the EANM

AI shows great promise in improving image quality, personalising dosages (both in diagnosis and theranostics) and helping in image interpretation and subsequent analysis. It opens up ways of fully exploiting the potential of NM, which has recently witnessed technological developments such as total-body PET, where the large amount of data acquired will also benefit from AI. NM has also strived to quantify molecular processes using PET and SPECT, and here again, AI may help with the process. As such, AI has the potential to improve clinical workflows that will increase overall efficiency but also facilitate personalised medicine for the benefit of individual patients. There remains, however, a long road ahead before the potential of AI in NM is realised in a manner acceptable to both healthcare professionals and patients. EANM can, and will, play a key role in this process.

### Defining unmet needs

EANM should help to define unmet needs in the operational, physics, and clinical fields. By working together as a community, we can identify the issues that are most pressing along with those that are most likely to benefit from AI. Thereafter, we will need to prioritise and define clear objectives. Oncology is currently top of the list of the most widely published applications of AI in NM, as shown by a systematic review in 2019 [17]. More specifically, 86% of all publications in the AI and radiomics field dealt with oncology [17]. Nonetheless, cardiology, as illustrated by a recent position paper [18], neurology, inflammation, and infection, as well as therapy, should all benefit from those developments. It is here that the different committees of the EANM, together with their counterparts in other societies, will have to take the lead.

### Setting standards

Second, we need to define the methods and set the standards against which the AI solutions will be evaluated and “calibrated”. This implies defining methodological details (DL algorithm architectures), statistics, and sample sizes used in the different stages of algorithm development, evaluation and validation, endpoints including ground truth, etc. The current literature is very heterogeneous in most of these aspects. More importantly, basic concepts such as the metrics used for measuring the performance of the models in the different targeted applications need to be clearly outlined [19]. For every solution, the different validation processes need to be clearly determined in advance. Harmonisation, transparency, and generalisability are key for a trustworthy clinical implementation. The development of similar initiatives to the “Image Biomarker Standardization Initiative” in the radiomics field will be a major step forward [20]. The EANM’s EARL Initiative could in time also become an important player, as it is familiar with standardisation aspects.

One of the difficulties with DL algorithms is not only the multiple parameters that need to be optimised but also the different types of networks and their implementation details in terms of, e.g. the number of layers and associated connections, optimisers, loss functions, etc. Each of these parameters can be varied. Consequently, it is crucial to compare the performance of all these various implementations under controlled conditions. Given all the potential variations, it is impossible to reproduce results from the literature by reimplementing the proposed developments. Instead, alternative approaches such as software challenges need to be implemented. In these challenges, a given dataset is made available for developers to evaluate the performance of their algorithms within a controlled environment. Numerous software challenges have been organised throughout the years, mainly within the field of image segmentation but also in other fields of interest and increasingly targeting clinical endpoints (https://grand-challenge.org/challenges/).

Software challenges should be based on a predetermined categorisation of potential applications, using different factors such as impact and potential utility and uptake in clinical practice. Challenges should address one or more of the following points:

1. Specify the need for data and associated requirements (volume, annotation, QC), defining training and validation datasets.
2. Clarify the dependency of data volumes necessary for a given task, which should also include standardisation and/or harmonisation for the exploitation of multicentre datasets.
3. Define algorithm-related aspects such as the number of parameters, their optimisation, robustness, levels of uncertainty, and transferability to datasets from different instruments or body locations.
4. Deal with model interpretability (white/grey box concept as opposed to black box), and thus with acceptability to professionals (medical physicists, physicians) and the public (patients and families).
5. Integrate established domain knowledge (e.g. PBPK modelling) in AI algorithms or training procedures to reduce data volume requirements and improve the robustness of the algorithms.
6. Consider training issues for implementation in clinical practice.

In order to accelerate clinical adoption, validation of the outcomes/results of individual trials/studies is required, and this is where multicentre and multigroup cooperation is of the utmost importance. Such cooperations within the field of NM can and should be triggered by challenges that link methodological and clinical objectives. A European platform such as the EANM with the expertise within its committees is ideally suited for overseeing such challenges, and it also represents a unique opportunity for the EANM to participate in the worldwide efforts of open science by facilitating pan-European collaborations.

### Increasing awareness and knowledge

We need to increase the overall level of knowledge and competence in all fields related to AI. This implies revamping the nuclear medicine curricula for both medical physicists and physicians to take account of this evolution. Computational sciences, in particular, must be further integrated into education and training [21]. Although training is the responsibility of each individual European state through their national requirements, societies like the EANM can help increase awareness of the ongoing (r)evolution and promote standardisation within Europe. For example, the next update of the “European Training Requirements” published by the Nuclear Medicine Section of the UEMS should reflect this new dimension and provide guidance as to the minimum content of AI-related matters in the training curriculum, including quality criteria for trainers and training sites. The EANM will play a leading role within the continuing education field, making sure that both its flagship Annual Conference and the education programmes run by ESMIT facilitate the transmission of knowledge for the appropriate and reasoned implementation of AI in NM. These educational programmes can target different AI-related aspects, be they scientific, clinical, or ethical. Such programmes should meet the requirements for high-risk systems set out in the European Artificial Intelligence Act, which state that no AI systems are to be approved without providing users with information on the capabilities and limitations of AI and how to use it.

The time has come to incorporate AI as another partner discipline. Revamping the training of NM physicians implies enhancing existing collaborations between medical physicists, engineers, and specialists in different clinical applications but also the development of closer cooperation with other scientific societies dedicated to medical image computing and analysis. The EANM may also play a key role in helping industrial partners target appropriate developments and associated clinical applications in our field. At the same time, it can contribute to initiatives coordinated by the EU in the context of the deployment of trustworthy AI. Such initiatives include the Artificial Intelligence Regulation, the Data Act, the Digital Governance Act, and the European Health Data Space.

### Ethical standards

Last but not least, ethical standards for the implementation of AI in our field and associated clinical applications need to be set out by the EANM. Currie et al. have recently proposed a set of ethical standards to be followed when evaluating and developing AI in NM as listed in Table 1 [22]. The EANM fully embraces these principles, which are applicable to any medical speciality that will employ AI as part of its clinical practice in the future. We believe they constitute a solid and necessary framework within which AI can be incorporated into nuclear medicine.

**Table 1:** Ethical standards to be followed when evaluating and developing AI in nuclear medicine (adapted from Currie et al.)

1	Beneficence, i.e. common good
2	Non-maleficence, i.e. do no harm
3	Fairness and justice, i.e. equal opportunity and access
4	Safety, i.e. for the patient
5	Reliability, i.e. accuracy and reproducibility in clinical practice
6	Security, i.e. for the data
7	Privacy and confidentiality of data
8	Mitigation of bias, i.e. fair and evidence-based clinical validation
9	Transparency and visibility, i.e. to the patients and community
10	Explainability and comprehensibility
11	Human values: human-in-the-loop process incorporated
12	Autonomy, judgement, and decision-making: human-in-the-loop process
13	Collegiality, i.e. multidisciplinary involvement and commitment
14	Accountability, i.e. among stakeholders
15	Governance, i.e. framework to ensure compliance
16	Inclusiveness, i.e. empowerment of all stakeholders

Many of those principles appear self-evident, albeit not equally so in all parts of the world. Yet, some are easier said than done. For instance, the “human-in-the-loop” process when making decisions regarding a diagnosis, and the shared accountability of all stakeholders in the implementation of AI solutions, raise questions when projected into the routine setting. Having a human in the loop is indeed all well and good, but what will happen if the AI system proves to be more trustworthy? Past experience with computer-aided diagnosis, which is a separate process from machine learning, showed troubling instances where radiologists largely ignored the correct computer prompts [23]. Again, at this stage, these are theoretical questions, but they ought to be considered part of any clinical implementation of such technology in our practice.

In conclusion, AI is here to stay, and with-it, NM will likely thrive in the foreseeable future. However, AI does not come without a cost. Several preconditions need to be met before AI is able to show its full potential. By its very nature, nuclear medicine is ready to adapt to the necessary changes, and the EANM will fully support the education on AI and the efficient implementation of AI at all levels in NM.

## References

[1] Visvikis D, Lambin P, Mauridsen KB, Hustinx R, Lassmann M, Rischpler C, et al. Application of artificial intelligence in nuclear medicine and molecular imaging: a review of current status and future perspectives for clinical translation. Eur J Nucl Med Mol I. 2022. 10.1007/s00259-022-05891-w. 10.1007/s00259-022-05891-w PMC9606092 35809090

[2] Kotter E, Ranschaert E (2021). Challenges and solutions for introducing artificial intelligence (AI) in daily clinical workflow. Eur Radiol..

[3] Lexa FJ, Jha S (2021). Artificial intelligence for image interpretation: counterpoint—the radiologist’s incremental foe. Am J Roentgenol..

[4] Huisman M, Ranschaert E, Parker W, Mastrodicasa D, Koci M, de Santos DP (2021). An international survey on AI in radiology in 1,041 radiologists and radiology residents part 1: fear of replacement, knowledge, and attitude. Eur Radiol..

[5] Ridley EL. ECR: AI adoption on the rise in the Netherlands [Internet]. 2022 [cited 2022 Jul 13]. Available from: https://www.auntminnieeurope.com/index.aspx?sec=rca&sub=ECR_2022&pag=dis&ItemID=622799.

[6] Hustinx R (2019). Physician centred imaging interpretation is dying out — why should I be a nuclear medicine physician?. Eur J Nucl Med Mol I..

[7] Zhou Z, Firestone C (2019). Humans can decipher adversarial images. Nat Commun..

[8] Barucci A, Neri E (2020). Adversarial radiomics: the rising of potential risks in medical imaging from adversarial learning. Eur J Nucl Med Mol I..

[9] European Commission. Regulatory framework proposal on artificial intelligence. Available from: https://digital-strategy.ec.europa.eu/en/policies/regulatory-framework-ai.

[10] Sibille L, Seifert R, Avramovic N, Vehren T, Spottiswoode B, Zuehlsdorff S (2019). 18F-FDG PET/CT uptake classification in lymphoma and lung cancer by using deep convolutional neural networks. Radiology..

[11] Pinochet P, Eude F, Becker S, Shah V, Sibille L, Toledano MN (2021). Evaluation of an automatic classification algorithm using convolutional neural networks in oncological positron emission tomography. Frontiers Medicine..

[12] Isensee F, Jaeger PF, Kohl SAA, Petersen J, Maier-Hein KH (2021). nnU-Net: a self-configuring method for deep learning-based biomedical image segmentation. Nat Methods.

[13] Iantsen A, Ferreira M, Lucia F, Jaouen V, Reinhold C, Bonaffini P, et al. Convolutional neural networks for PET functional volume fully automatic segmentation: development and validation in a multi-center setting. Eur J Nucl Med Mol I. 2021:1–13. 10.1007/s00259-021-05244-z PMC8440243 33772335

[14] Hatt M, Laurent B, Ouahabi A, Fayad H, Tan S, Li L (2018). The first MICCAI challenge on PET tumor segmentation. Med Image Anal..

[15] Pianykh OS, Langs G, Dewey M, Enzmann DR, Herold CJ, Schoenberg SO (2020). Continuous learning AI in radiology: implementation principles and early applications. Radiology..

[16] European Commission. Proposal for a Regulation of the European Parliament and of the Council laying down harmonised rules on artificial intelligence (Artificial Intelligence Act) and amending certain union legislative acts. 2021. Available from: https://eur-lex.europa.eu/legal-content/EN/TXT/?uri=CELEX%3A52021PC0206.

[17] Sollini M, Antunovic L, Chiti A, Kirienko M (2019). Towards clinical application of image mining: a systematic review on artificial intelligence and radiomics. Eur J Nucl Med Mol I..

[18] Slart RHJA, Williams MC, Juarez-Orozco LE, Rischpler C, Dweck MR, Glaudemans AWJM (2021). Position paper of the EACVI and EANM on artificial intelligence applications in multimodality cardiovascular imaging using SPECT/CT, PET/CT, and cardiac CT. Eur J Nucl Med Mol I..

[19] Erickson BJ, Kitamura F (2021). Magician’s Corner: 9. Performance metrics for machine learning models. Radiology Artif Intell..

[20] Zwanenburg A, Vallières M, Abdalah MA, Aerts HJWL, Andrearczyk V, Apte A (2020). The image biomarker standardization initiative: standardized quantitative radiomics for high-throughput image-based phenotyping. Radiology..

[21] Xing L, Goetsch S, Cai J (2021). Artificial intelligence should be part of medical physics graduate program curriculum. Med Phys..

[22] Currie G, Hawk KE, Rohren EM (2020). Ethical principles for the application of artificial intelligence (AI) in nuclear medicine. Eur J Nucl Med Mol Imaging..

[23] Nishikawa RM, Schmidt RA, Linver MN, Edwards AV, Papaioannou J, Stull MA (2012). Clinically missed cancer: how effectively can radiologists use computer-aided detection?. Am J Roentgenol..

